# Gratuities of Physicians

**Published:** 1885-12-20

**Authors:** 


### GRATUITIES OF PHYSICIANS.

It may be truthfully asserted that Physicians do more gratuitous work than any other class of citizens. The nature of the profession is such that they are constrained to bestow their time and labor for the relief of the indigent sick without hope of compensation, and, often, without the thanks of the parties thus favored. There are two powerful forces acting upon the Physician which impels him to do this gratuitous work: the one is the force of public sentiment, which will not allow to the Physician the same liberty to decline these services as exists with those of other callings. The merchant may refuse to credit whom he will, and the lawyer can make arrangement for security of his fee in advance of the service to be rendered; but such action on the part of the Physician would incur the displeasure and condemnation of the entire community, and jeopardize or destroy his business prospects. Another force acting upon the Physician to impel him to these charities is that of Humanity, which, to the conscientious man, is even stronger than the unreasonable exactions of public sentiment.

Some years ago the Medical Association of Georgia memorialized the Legislature to relieve Physicians of the special tax of $10 which is imposed upon them by the State, urging these and other strong reasons for such exemption; but the memorial failed of its object. Among the arguments then presented to the Legislature was the great quantity of medicines given away by Physicians to the poor, aggregating hundreds of thousands of dollars per annum, in the State, and which ought to be supplied by the public and not by the Physician, who must carry also this burden in addition to the time, labor and care which he gratuitously bestows. To say that these charities are incident to his calling, and must be endured, is illiberal and contemptible on the part of the Legislature of a great State.

But we do not propose here to detail the many arguments touching the illiberality of the State, and of the community, towards Physicians, or the injustice of the special tax by the State.

There is one minor point to which our attention has been directed, to wit, "Ought doctors to charge preachers?" To this we will simply say, what we think the rule ought to be in this case: the preacher who is getting a living salary ought to pay his physician like any other men, and we have a poor opinion of a minister who will accept gratuitous services of his physician, especially if he is getting a good salary—and many of them get salaries, which to the great majority of doctors would be a princely income. There are, of course, instances where the doctor should not charge the minister, and of these he is very properly his own judge. The truth is, that medical men, as a class, are more liberal and self-sacrificing than any other, and we are glad of it; but the public are becoming more and more exacting and unreasonable toward the profession, and seem now disposed to demand as a right what the profession in its generosity has been wont to bestow as a favor.

